# Impacts of Longer-Term Exposure to AuNPs on Two Soil Ecotoxicological Model Species

**DOI:** 10.3390/toxics10040153

**Published:** 2022-03-22

**Authors:** Bruno Guimarães, Susana I. L. Gomes, Janeck J. Scott-Fordsmand, Mónica J. B. Amorim

**Affiliations:** 1 Department of Biology & Centre for Environmental and Marine Studies (CESAM), University of Aveiro, 3810-193 Aveiro, Portugal; bguimaraes@ua.pt (B.G.); susana.gomes@ua.pt (S.I.L.G.); 2 Department of Ecoscience, Aarhus University, Vejlsovej 25, P.O. Box 314, DK-8600 Silkeborg, Denmark; jsf@ecos.au.dk

**Keywords:** terrestrial ecosystem, persistent pollutants, metallic nanoparticles, long-term, *Enchytraeus crypticus*, *Folsomia candida*

## Abstract

The production, use and disposal of nanoparticles (NPs) has been increasing continuously. Due to its unique properties, such as a high resistance to oxidation, gold NPs (AuNPs) are persistent in the environment, including the terrestrial, one of the major sinks of NPs. The present study aimed to assess the effects of AuNPs (from 10 to 1000 mg/kg) on two OECD standard ecotoxicological soil model species, *Enchytraeus crypticus* and *Folsomia candida*, based on the reproduction test (28 days) and on a longer-term exposure (56 days), and survival, reproduction, and size were assessed. AuNPs caused no significant hazard to *F. candida*, but for *E. crypticus* the lowest tested concentrations (10 and 100 mg AuNPs/kg) reduced reproduction. Further, AuNPs’ toxicity increased from the 28th to the 56th day mainly to *F. candida*, as observed in animals’ size reduction. Therefore, longer-term exposure tests are recommended as these often reveal increased hazards, not predicted when based on shorter exposures. Additionally, special attention should be given to the higher hazard of low concentrations of NPs, compared to higher concentrations.

## 1. Introduction

The production of engineered nanoparticles (NPs) has increased exponentially in the past decades. Soil is the major sink of NPs’ release into the environment [1], in some cases with severe release, e.g., 20,000 tons/year for TiO_2_NPs [2]. It is thus not surprising that, despite the analytical difficulties of measuring NPs in complex natural matrices, NPs have been detected in various environmental media [1,3]. Hence, due to their extensive use and environmental persistence, metal-based NPs pose particular concern for environmental safety [4].

Among the metallic NPs, gold nanoparticles (AuNPs) are used for a wide range of applications, e.g., in catalysts, electronics, paints, cosmetics, cancer treatments, molecular biology research and biomedical applications [5,6]. The mean annual predicted environmental concentrations (PEC) of AuNPs in the soil ecosystem is 0.0059 mg/kg in the UK [7]. PEC in sewage sludge are 0.12 and 0.15 mg/kg for UK and US, respectively, and in sludge-treated soil up to 0.3 and 0.15 mg/kg for UK and US, respectively [8]. Due to the extremely high resistance to oxidation, Au metal will remain insoluble under ambient conditions [9]. Therefore, despite the fact that these concentrations can be considered low, AuNP concentrations are expected to increase in the environment [10].

It is well shown that AuNPs can be taken up by organisms [11,12], and may even show trophic transfer. Such Au uptake has been shown to cause both acute or longer-term toxicity, e.g., as shown in in vitro studies, bacteria and in vivo in higher organisms [12,13,14,15,16,17,18,19,20]. There is still lack of knowledge, especially regarding soil invertebrates. Available information on AuNPs in soils includes, e.g., in enchytraeids (causing no effects after 14-days exposure to *Enchytraeus buchholzi* [21]), earthworms (triggering oxidative stress and DNA modifications [22] and reducing reproduction [23] in *Eisenia fetida*, and reducing survival and reproduction of *Eudrilus eugeniae* after 50-days exposure [24]), nematodes (altering gene expression [25] and reducing reproduction [26,27] in *Caenorhabditis elegans*) and isopods (with no effects on *Porcellio scaber* [10]).

We are particularly interested here in the longer-term effects, as studies with other NPs have shown that standard exposure duration tests underestimated the toxicity of NPs observed after prolonged and/or multigenerational exposures. For example, multi-walled carbon nanotubes (MWCNTs) [28] and tungsten carbide cobalt (WCCo) NPs [29] induced a significant population decrease in *Enchytraeus crypticus* after an extended exposure period of 60 days, although there was no impact after the standard 28 days. Moreover, a multigenerational exposure to WCCo [30] reported no significant impact in terms of survival and reproduction at 1500 mg/kg, but a skewed size distribution was observed with relatively more large (and medium), rather than small, organisms. Other examples include silver (Ag) NPs (AgNM300K) where toxicity was higher in *E. crypticus* based on the full life cycle test (46 days), in comparison to the standard enchytraeid reproduction test (ERT) [31].

In this study we aimed to assess the toxicological effects of AuNPs stabilized with polyvinylpyrrolidone (PVP) over longer-term exposure (56 days), in comparison to the standard (28 days), on survival, reproduction, and size of *E. crypticus* and *F. candida*. The AuNPs tested are part of the repository of test materials within the EU H2020-NMBP-2017 BIORIMA project, as a nano-biomaterial (NBM) with potential applications as antimicrobial, X-ray contrast agent, and in photodynamic therapy. Besides being model species in soil ecotoxicology [32,33], *E. crypticus* and *F. candida* have similar standardized one generation exposure time (around 28 days), and both also have well-developed longer-term tests (56 days duration) [29,34]. This allows for a comparison between species at similar exposure periods.

## 2. Materials and Methods

### 2.1. Test Organisms

The standard test species *Enchytraeus crypticus* (Oligochaeta: Enchytraeidae) and *Folsomia candida* (Collembola) were used. *E. crypticus* cultures were maintained at 20 ± 1 °C, under a photoperiod of 16:8 (light:dark), in petri dishes with agar, consisting of sterilized Bacti-Agar medium (Oxoid, Agar No. 1) and a mixture of four different salt solutions at the final concentrations of 2 mM CaCl_2_·2H_2_O, 1 mM MgSO_4_, 0.08 mM KCl, and 0.75 mM NaHCO_3_. Individuals were fed with ground autoclaved oats twice per week. Synchronized cultures were prepared as detailed in Bicho et al. [35], and juveniles (17–18 days) were used. *F. candida* organisms were cultured at 20 ± 1 °C, under a photoperiod of 16:8 (light:dark), on a moist substrate of plaster of Paris and activated charcoal (8:1 ratio). Individuals were fed weekly with dried baker’s yeast (*Saccharomyces cerevisiae*). Cultures were synchronized to obtain 10–12 days old juveniles.

### 2.2. Test Soil

The natural standard LUFA 2.2 soil (Speyer, Germany) was used for the experiments and is characterized as follows: pH (0.01 M CaCl_2_): 5.6 ± 0.4; organic carbon: 1.71 ± 0.30%; cation exchange capacity (CEC): 9.2 ± 1.4 meq/100 g; maximum water holding capacity (WHCmax): 44.8 ± 2.9 g/100 g; texture: 8.0 ± 1.5% clay, 13.7 ± 1.0% silt, and 78.3 ± 1.0% sand content.

### 2.3. Test Material, Characterization and Spiking Procedures

A AuNPs aqueous suspension, stabilized with PVP (provided by Colorobbia, BIORIMA, Cookeville, TN, USA) was used, plus the respective dispersant alone. The AuNPs dispersion was prepared as follows: an aqueous solution of Na_3_Cit was added to an aqueous solution of PVP heated to 80 °C, followed by the quick addition of a solution of HAuCl_4_; after cooling down, the suspension was then dialyzed with deionized water to remove excess PVP and citrate with a Tangential Flow membrane (Pellicon XL 10kDa PES). Particles were ca. 40 nm in diameter and negatively charged (ξ-potential = −14.4 mV); peak one size is 37.9 nm and peak two is 3.1 nm, with a Z-average of 27.7 nm (see Table S1 for characterization).

The tested concentrations were 0–10–100–200–1000 mg Au/kg for the AuNPs and the equivalent to 1000 mg Au/kg for the dispersant control (i.e., using the same volume). The selection of concentration range aimed to capture a dose–response curve for modelling purposes, naturally above PEC, since Au has been shown not to be very toxic. Spiking followed the OECD recommendations for nanomaterials in solution [36]. The as provided stock aqueous dispersions were serially diluted, and the correspondent volumes were applied with a micropipette onto the pre-moistened soil and homogeneously mixed with a stainless-steel spatula for ca. 3 min. Spiking was carried out per individual replicate. Moisture was adjusted to 50% of the soils’ WHCmax and soil was allowed to equilibrate for 1-day prior to test start.

### 2.4. Test Procedure

Tests with enchytraeids followed the standard guideline for the Enchytraeid Reproduction Test (ERT, 28 days) [33], plus the OECD extension, as described in, e.g., Ribeiro et al. [29]. In short, the test was extended or more days (56 days in total) and extra monitoring sampling times were added at days 7, 14, 21, (28) and 56. Endpoints included survival for all sampling periods and reproduction at days 28 and 56, i.e., number of juveniles and population, respectively. Number of replicates per treatment were one at days 7, 14 and 21, and four at days 28 and 56.

At test start, ten synchronized age organisms (18–20 days old after cocoon laying) were introduced in each test vessel with moist soil (⌀4 cm with 20 g of soil for exposure up to day 28, and ⌀5.5 cm with 40 g of soil for exposure up to day 56) and food supply (22 ± 2 mg, autoclaved rolled oats). Test ran up to 56 days at 20 ± 1 °C and 16:8 h photoperiod. Food (11 ± 1 mg: until day 28, and 33 ± 3 mg: from 28 to 56 days) and water were replenished weekly. On sampling days 7, 14, 21, and 28, adults were carefully removed from the soil and counted (survival). The juveniles were counted at day 28 and 56 using a stereo microscope, to assess reproduction. After being fixed for 24 h with ethanol and stained with Bengal rose (1% in ethanol), soil samples were sieved through meshes with decreasing pore size (1.6, 0.5, and 0.3 mm) to separate the enchytraeids from most of the soil and facilitate counting. For the replicates that continued until day 56, adults were carefully removed from the soil at day 28.

Test with collembolans were performed using the standard guideline OECD 232 [32] (28 days), plus the OECD extension, as described in, e.g., Guimarães et al. [34]. In short, the test was extended for 28 more days (56 days in total) with adding extra monitoring sampling times at days 7, 14, 21, (28) and 56 days. Endpoints included survival and reproduction, i.e., number of juveniles (or population at day 56). Size of organisms was assessed at days 28 and 56. Four replicates per treatment were performed.

At test start, ten synchronized age animals (10–12 days old) were placed in each test vessel with moist soil (⌀5.5 cm, 30 g of soil) and food supply (2–10 mg, baker’s yeast). Test ran up to 56 days at 20 ± 1 °C, under a 16:8 h photoperiod. Food and water were replenished weekly. At each sampling day (7, 14, 21, 28 and 56 days), the test vessels were flooded with water, the content was transferred to a crystallizer dish and the surface was photographed for further analyses (count and measure (size, area)) using the software ImageJ [37]. For the replicates that continued until day 56, after a similar flooding and photographing procedure, the sampled juveniles at day 28 were transferred with a spoon to a box with a layer of Plaster of Paris to absorb extra water from the spoon. After this, ten of the biggest juveniles (ca. 11 days old) were transferred to new test vessels containing soil (spiked at day 0), representing the F1 follow-up exposure, and the test ran under the same exact conditions. At day 56, survival (F1) and reproduction (F2) were counted and measured, following the previously described procedure.

Soil pH (0.01 M CaCl_2_) was measured at test start (day 0) and at days 28 and 56 on both species’ tests.

### 2.5. Data Analysis

One-way analysis of variance (ANOVA), followed by the Dunnet’s post-Hoc test was used to assess differences between control and treatments for all the endpoints (survival, reproduction and size), and between exposure time (28 and 56 days) [38].

## 3. Results

**Figure 1:**
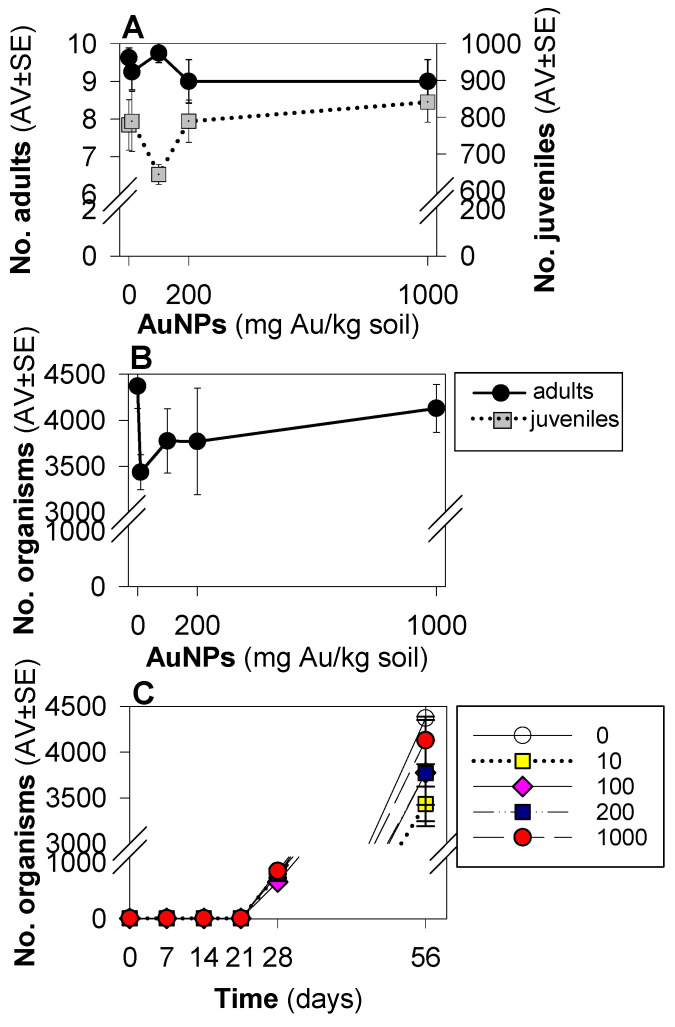
Results of the reproduction test with *Enchytraeus crypticus* when exposed in LUFA 2.2 soil to gold nanoparticles (AuNPs), over 56 days, in terms of number of adults and juveniles at day 28 (**A**), total number of organisms at day 56 (**B**), and in terms of total number of organisms at days 0, 7, 14, 21, 28 and 56 of exposure (**C**). Values are expressed as average ± standard error (AV ± SE).

Results from the standard enchytraeid reproduction test (Figure 1) showed that the test validity criteria were fulfilled, i.e., in controls adult mortality was 7.5% (<20%), number of juveniles was 675 (>50), and coefficient of variation was 29% (<50%). There were no significant differences between the water and dispersant controls, hence they were polled for graphical representation and statistical analysis. No significant change in pH was observed (full details in Table S2).

Survival and reproduction were not significantly affected by AuNPs’ exposure at either 28 (standard) or 56 days (extension) (Figure 1). However, for *E. crypticus* the lowest tested concentrations (10 and 100 mg AuNPs/kg) caused a reduced population number (reproduction) after 56 days (Figure 1B). It is further noted that 100 mg AuNPs/kg also caused a drop in reproduction at day 28 (Figure 1A).

**Figure 2:**
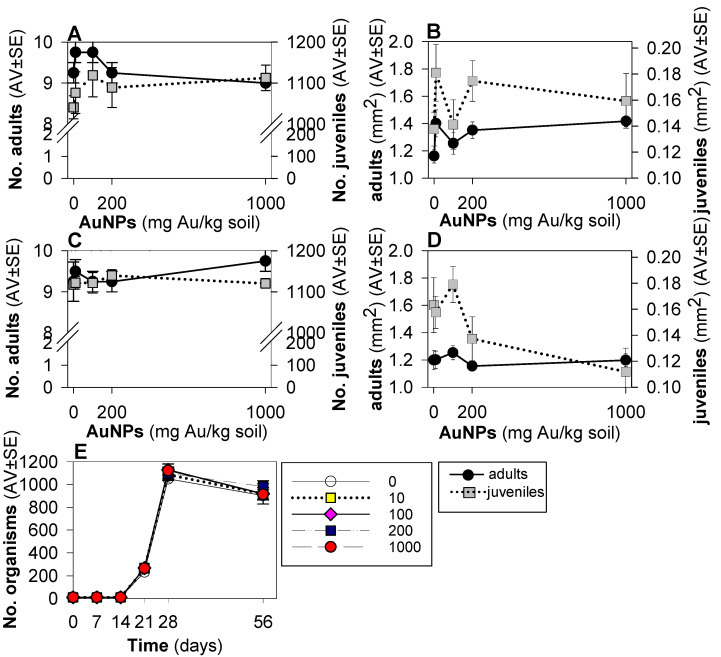
Results of the reproduction test with *Folsomia candida* when exposed in LUFA 2.2 soil to gold nanoparticles (AuNPs), in terms of number of adults, juveniles and size at day 28 (**A**,**B**), at day 56 (**C**,**D**), and in terms of total number of organisms at days 0, 7, 14, 21, 28 and 56 of exposure (**E**). Values are expressed as average ± standard error (AV ± SE).

Results from the standard reproduction test with *F. candida* (Figure 2) showed that the test validity criteria were fulfilled i.e., in controls mortality was 7.5% (<20%), number of juveniles was 1041 (>100), and coefficient of variation was 5.4 (<30%). There were no significant differences between the water and dispersant controls, hence the replicates were polled for graphical and statistical analysis. No significant change in pH was observed (Table S2).

For the standard exposure (28 days), survival, reproduction and size were not affected by AuNPs (Figure 2). For the extension (56 days) exposure, again no effects were observed on survival or reproduction, but juveniles showed a tendency to be smaller from 200 mg/kg, although not significantly (Figure 2D). Comparing results from day 28 and 56, the number of organisms showed a general decrease (although not significant) for all treatments (Figure 2E).

## 4. Discussion

No significant concentration–response effects of AuNPs were observed on survival or reproduction for both *E. crypticus* and *F. candida*, at concentrations up to 1000 mg Au/kg soil. However, the intermediate concentration (10 and 100 mg AuNPs/kg) caused a decrease in reproduction in *E. crypticus*. This we have observed before for other NPs, e.g., Ag [31,39] and Ni [40]. In the case of *F. candida*, there was approximately 30% size reduction at the highest concentration (1000 mg/kg, 56 days exposure), in what appeared to be a concentration–response course. This smaller size indicates longer-term population risk, because animals are known to require a minimum size to reproduce [41,42].

AuNPs have previously been reported as non-toxic to other soil invertebrate species. For instance, in *E. buchholzi*, Patricks et al. [21] reported no effects of 14 days exposure to AuNPs on survival and reproduction up to 37.5 mg AuNPs/kg soil (which is obviously lower than our exposure concentrations). Similarly, no effects were observed for *P. scaber* mortality, body mass, or food consumption when exposed through food [10] during 14 days to 10 and 60 mg AuNPs/kg (cannot be directly converted to soil concentrations). A study with the earthworm *Eudrilus eugeniae*, exposed to AuNPs for 50 days, reported no mortality, but the worms were found to be less active after the 10th day [24] (here the exposure concentration is unclear, as it is stated as 10 mg Au/box).

Other studies have shown that AuNPs can cause toxicity, e.g., while exposure of *E. fetida* to 5, 20 and 50 mg Au/kg of AuNPs (20 and 55 nm) for 28 days did not affect survival or growth, it did cause a decreased reproduction at 50 mg/kg (for the smaller AuNPs) and at 20 mg/kg (for the larger AuNPs) [23]. The authors of [23] showed that AuNPs accumulation in earthworms was concentration dependent for the larger NPs (55 nm), but not for the smaller (20 nm) (with higher AuNPs internalization for lower doses). It was hypothesized that this could be due to a high level of aggregation of the 20 nm particles to larger than 150 nm units in the higher concentration, whereas the 55 nm AuNPs remained mono-dispersed [23]. On the other hand, for *C. elegans*, it was observed that smaller (11 nm) citrate-stabilized 100 μg AuNPs/mL induced a significant decrease in the survival and reproduction, whereas exposure to larger (150 nm) caused no significant effects [43]. Here, the authors reported that the higher uptake of the smaller AuNPs (ca. 500 times more than the larger) was the main responsible for their higher toxicity [43]. It is well known from other NPs that uptake and toxicity depend on size and concentration, which also influence the agglomeration pattern [44,45].

As to our experiments, the decrease in *E. crypticus* reproduction at the lower concentrations (10 and 100 mg AuNPs/kg) could be due to the higher dispersibility, and with a resulting enhanced availability and related toxicity. An alternative or concurrent explanation could be that the size of the mouth (i.e., diameter in the opening) of the *E. crypticus* adults is ca. 100 μm [46], hence ingestion of the AuNPs would only be possible for homo- or hetero-aggregated NPs up to 100 μm (and possibly only the smaller NPs would facilitate uptake through the animals’ gut epithelium, limiting the toxicity of larger particles). This has been suggested before in *F. candida*, which was unaffected by a red organic pigment NM exposure, but it was clear that the pigment was ingested, shown by the visible color on the animal’s midgut [47]. *F. candida’s* mouth is also ca. 100 μm [48], but being a detritivore, this species can fractionate food before ingesting it. Hence it could be hypothesized that *F. candida* is able to ingest materials of larger sizes, compared to *E. crypticus*. The above considerations of dispersibility and mouth sizes could be explanatory factors for the impact, i.e., reduction of the size of the juveniles, at the highest tested concentration (1000 mg/kg) for *F. candida*. However, is it obvious that 100 µm is quite large and many soil particles are below this threshold, hence for such hetero-aggregates this would not hold.

On the other hand, when the same AuNPs were combined with activated sludge and added to soil [49], *E. crypticus* reproduction increased while decreasing the organism’s size after 56-days exposure, whereas for *F. candida* there was a minor decrease of the reproductive performance and size. As stated above, a smaller size for juveniles indicates an increased population risk, because animals are known to require a minimum size to reproduce [41,42]. When organisms are smaller, they take longer to mature (that is if they make it to maturity within a season), and this may affect present and subsequent generations.

Hence, this suggests the importance of longer-term exposure, e.g., as multigenerational exposure, especially for persistent materials which exert chronic effects. In line with this, a multigeneration study with *C. elegans* reported that the severity of reproductive toxicity of AuNPs in *C. elegans* was directly related to increased exposure time [26]. A decrease in the number of produced eggs after exposure to 100 μg/mL of AuNPs (11 nm) from F0 was detected, this effect being observed at lower concentrations (10 and 50 μg/mL) from F2 [26]. Another multigenerational study [50] showed that the effects of AuNP exposure for one generation in *C. elegans* (parental generation) can induce adverse effects on subsequent generations’ reproduction, i.e., transgenerational effects. Finally, Moon et al. [27] showed that continuous exposure of AuNPs to *C. elegans* impaired reproduction from F2 to F4, while intermittent exposure caused more severe effects on F3 organisms, which may indicate that the recovery period was not sufficient for the animal.

From a wider perspective, among NPs, the AuNPs were less toxic (mass based) to *E. crypticus* than other metallic NPs e.g., CuO NMs [51] and WCCo [29], at comparable exposure periods. For *F. candida*, the toxicity of AuNPs was also lower (mass based) than reported for other metallic NPs, e.g., AgNPs [39] and ZnONPs [52]. Although some NPs such as WCCo, CuO, organic pigment and MWCNTs, showed no toxicity to *F. candida* in the standard 28 days reproduction test [47], in multigenerational exposure (four generations) WCCo NM affected reproduction and survival from F3 onwards, while CuO NM caused no effects [53].

## 5. Conclusions

Hazards caused by AuNPs for soil invertebrates seem to be concentration and exposure time dependent. For *E. crypticus* the lowest concentration caused a reduction in reproduction for both standard (28 days) and extension (56 days) exposures (100 mg AuNPs/kg at day 28 and 10 mg AuNPs/kg at day 56). For *F. candida*, the size of animals was reduced at the highest concentration (1000 mg AuNPs/kg) after an extension exposure (56 days) period. Given the high persistency of AuNPs, they remain in the environment for long periods of time, and hence can accumulate to higher levels. Therefore, longer-term exposure tests are recommended, including multi-generational tests to assess the risks of AuNPs to soil invertebrates. Moreover, special attention should be given to the potential higher hazard of low concentrations of NPs, including AuNPs, compared to higher concentrations.

## Data Availability

The data presented in this study are available on request from the corresponding author.

## Supplementary Materials

The following supporting information can be downloaded at: https://www.mdpi.com/article/10.3390/toxics10040153/s1, Table S1. Characterization of the water suspension of gold nanoparticles (AuNPs), stabilized with polyvinylpyrrolidone (PVP) used in the experiments. DLS: dynamic light scattering; ICP-OES: inductively coupled plasma optical emission spectrometry; UV-Vis: Ultraviolet–visible spectroscopy; wt: weight; Pd: Polydispersity; kcps: count rate; σ: standard deviation; n/a: not available, Table S2. Variation of the soil pH (0.01 M CaCl_2_) per test condition, species and exposure time (day 0, 28 and 56).
