# Supplementary material for: Impacts of Longer-Term Exposure to AuNPs on Two Soil Ecotoxicological Model Species
Source: Toxics. 2022 Mar 22;10(4):153. doi: 10.3390/toxics10040153 (PMC9032579; doi:10.3390/toxics10040153)
Supplement: Supplementary file 1 [file toxics-10-00153-s001.zip › toxics-1632920-supplementary.pdf]

toxics MDPI

Supplementary Materials: Impacts of Longer-Term Exposure to AuNPs on Two Soil Ecotoxicological Model Species

Bruno Guimarães, Susana I. L. Gomes, Janeck J. Scott-Fordsmand and Mónica J. B. Amorim

Table S1. Characterization of the water suspension of gold nanoparticles (AuNPs), stabilized with polyvinylpyrrolidone (PVP) used in the experiments. DLS: dynamic light scattering; ICP-OES: inductively coupled plasma optical emission spectrometry; UV-Vis: Ultraviolet–visible spectroscopy; wt: weight; Pd: Polydispersity; kcps: count rate; σ: standard deviation; n/a: not available.

**Characteristics**					**Technique**
**Complete formulation**	Gold (Au 0,54 % wt)<br>PVP (0,27 ± 0,5 %wt)				ICP-OES<br>gravimetric
**Dispersant medium**	Water solution; Sodium citrate: 1 mM				n/a
**Z-average** (nm)	27.69				DLS
**Pd Index**	0.276				DLS
**Polydispersity** (nm)	14.5				DLS
**Polydispersity** (%)	52.5				DLS
**Derived kcps**	127.6				DLS
**ξ-potential** (mV)	−14.4 ± 2.4				DLS
**Absorption peak** (nm)	525				UV-Vis
	**Size Distribution results**				
	Size (d.nm)	**% Int**	**σ**	**%Pd**	
**Peak 1**	37.90	95.0	15.06	39.7	DLS
**Peak 2**	3.091	5.0	0.7931	25.7	DLS

Table S2. Variation of the soil pH (0.01 M $CaCl_2$) per test condition, species and exposure time (day 0, 28 and 56).

**Concentration (mg/kg)**	*E. crypticus*			*F. candida*		
	Day			Day		
	**0**	**28**	**56**	**0**	**28**	**56**
**0**	6.2	5.72	5.41	5.25	5.15	4.69
**dispersant**	6.21	5.96	5.37	5.02	5.05	4.46
**10**	6.27	6.49	6.39	4.97	5.12	4.57
**100**	6.25	6.38	6.41	4.94	5.21	4.78
**200**	6.27	6.34	6.38	4.95	5.21	5.05
**1000**	6.24	6.25	6.11	4.91	5.22	5.08
